# Mycetismus: a review

**DOI:** 10.1093/gastro/gov062

**Published:** 2015-12-04

**Authors:** Michael R. Smith, Robert L. Davis

**Affiliations:** ^1^Department of Surgery, St. Barnabas Hospital, Bronx, NY, USA and; ^2^Department of Surgery, Lutheran Medical Center, Brooklyn, NY, USA

**Keywords:** amanita phalloides, mushroom poisoning, amatoxin, amanitin, vitamin C, cimetidine, silibinin, N-acetylcysteine, liver transplantation, Ganzert criteria, King’s College criteria, mycetismus, Clichy criteria.

## Abstract

Although rare, death from amanitin exposure poses a significant health risk and a diagnostic challenge to the clinician due to its rarity. This is one of the few conditions to be voluntarily reported by healthcare professionals. No antidote exists for this poisoning and, perhaps due to its rarity or lack of attention, the United States has lagged behind Europe for almost three decades in treatment, diagnostics and experimentation. This regrettable fact warrants the formation of a centralized agency for education, the advancement of research and the collection of data, to provide better treatment for the population.

## Introduction

Within most westernized countries, mycetismus is an unusual entity to encounter and approximately 50 to 100 cases are reported in America yearly, while in some European countries the number may be double this [1]. In countries where mushroom collection is common, such as Turkey, mycetismus may constitute the majority of toxic plant exposures [2]. The majority of the effects seen from the deadlier species of mushroom will not be noticed until days after ingestion. The first recorded case of mushroom poisoning in America occurred in 1871 but, since this time, only sporadic reports in the literature—and voluntary reports to local agencies—have been seen [3]. Despite the potential morbidity and mortality associated with these poisonings, no central agency has been created for the purposes of recording, cataloging, or producing a sustainable algorithm for treatment by randomized, controlled trials. In their 1977 book on the taxonification of mushrooms, Karen and Richard Haard comment on their frustration at this lack of a central agency. They further comment that, although there may be 50 or more deaths from mushroom poisoning yearly, most of these go unreported [4].

The current research and recommendations on the treatment of mushroom poisonings, specifically amatoxin, will be reviewed here. Our hope is not only that the deadly poisonings will be reported, but that all cases of mushroom poisoning, including those caused by the hallucinogenic types, will be mandatorily reported and information collected by a single United States agency. The goal of this paper is to raise awareness of the critical need for a central agency to monitor, educate, and increase the research into antidotes for mushroom poisonings. The amount of poisonings reported annually has been relatively unchanged for over fifty years; mushroom poisonings have been reported voluntarily to the Center for Disease Control and Prevention (CDC), North American Mycological Association (NAMA), and various state institutions and mycological societies. It is estimated that 10% of all symptomatic poisonings and approximately 50% of the deaths from mushroom poisonings are reported [5]. It is hoped that, from the evidence provided, a recognized national group will be established to meet the aforementioned purposes.

Of the 5000 known species of mushrooms, there are only approximately one hundred known to be poisonous. Their toxicities range from gastrointestinal irritants to those with psychotropic properties, while some cause acute renal or hepatic failure [6]. In the United States and Europe, it is estimated that 100–200 poisonings annually are associated with amatoxin poisonings, accounting for 90% of all fatal mushroom poisonings in those regions [7]. Mushroom poisonings are generally classified by their symptomatology, with those of the amatoxin family being commonly classified into the phalloides syndrome, which will be thoroughly described below [8]. The timing between ingestion and onset of symptoms is an important question in the historical interview of the patient, to determine the possible causative organisms [9]; however, when a sample of the causative organism is available, either from emetic material or remaining from prior to ingestion, an experienced toxicologist could determine the causative agent [4, 10]. It should be noted that foraging for mushrooms in the area where the ingested fungi were harvested is fruitless, as poisonous and non-poisonous mushrooms grow in immediate proximity to one another [9]. Most non-lethal mushrooms produce symptoms early after ingestion, with a short latency period of 30 to 60 minutes. It should be remembered that, even though the symptoms occur early, the possibility that a multitude of wild mushrooms were ingested should not exclude the possibility of a fatal ingestion [4, 9]. In patients who develop jaundice after an episode of gastrointestinal symptoms, the possibility of toxic mushroom ingestion should be moved to the forefront of the differential diagnosis [6].

## Significance

### Toxicology

The most infamous of mushroom poisons are the amatoxins (amanitins), most commonly derived from species of Amanita, their common names originating in the problems produced by their ingestion [11], e.g. *Amanita virosa *‘the destroying angel’ and *Amanita phalloides* ‘death cap’ [1]. Amatoxins are potentially fatal substances that are found as alpha (α) and beta (β) forms of dicyclic polypeptides [8, 13]. Amatoxins inhibit DNA-dependent ribonucleic acid (RNA) polymerase II, resulting in failure of protein synthesis and leading to injury in the most metabolically active tissues [9, 11]; thus the liver, the gastrointestinal tract and the convoluted tubules in the kidney are readily injured after administration of amanitin [7, 12]. Histological examination of hepatocytes reveals the abnormal presence of lipids and carbohydrates within the nucleus [9].

The toxin is readily absorbed by the intestinal epithelium by the OATP1B3 receptor and disappears rapidly from the plasma as it is weakly bound by plasma proteins. Forty-eight hours after ingestion, amatoxins are cleared from the plasma; however the toxin may remain in the enterohepatic circulation for up to four days [7, 14]. Sixty percent of the α-amatoxin is excreted into the enterohepatic circulation, prolonging the exposure of liver cells to the toxin as the cycle of absorption, excretion and re-absorption persists.

Amatoxin is nephrotoxic, causing injury to the proximal and distal convoluted tubules [9, 10]. The toxin is excreted into the urine and may be detectable for up to three to four days after ingestion [8]. The level in urine is usually higher than that found in the serum, but does not correlate to the degree of hepatonecrosis [10, 15]. The lethal dose of amatoxin for adults is 0.1 mg/kg [11]. As fresh mushroom tissue may have between 0.2 and 0.4 mg, ingestion of less than 50 g of fresh mushrooms may be lethal [8]. To put this in better perspective, as Reichl states, “Since 100 g of fresh amanita mushrooms contains up to 17 mg amanitin, eating a single mushroom can be fatal” [11]. Amatoxin is not heat-labile and neither cooking nor drying has an effect on the ultimate results of the ingested material [6, 10, 16].

### Clinical findings

The toxicity of amatoxin has been well studied and occurs in four phases. The initial phase is a latent period lasting 6 to 24 hours after ingestion, where the amatoxin is actively destroying the hepatic and renal cells. However, the patient generally experiences no discomfort as it is not irritating to the gastro-intestinal system [9, 13]. This phase averages ten hours, but may be as short as six hours, while documented cases of forty hours exist [7]. Although diarrhea usually occurs during the second phase of the illness, Escudie *et al*. have shown that an early predictor of a fatal outcome was the onset of diarrhea within eight hours of ingestion [12, 15].

Abdominal pain, nausea, vomiting, and diarrhea occur during the second stage of poisoning—the gastrointestinal phase—occurring 24–48 hours after ingestion. These symptoms may be accompanied by fever, tachycardia, hypoglycemia, hypotension, dehydration and severe electrolyte imbalance [9, 13]. This gastrointestinal phase usually occurs with grossly bloody diarrhea and emesis. Kidney function and hepatic function tests are usually within normal limits at this stage of illness [7].

The third stage—apparent convalescence—consists of a 24-hour period in which the patient appears to be improving, although continued evidence of renal and hepatic destruction will be evident from serum chemistries and hepatic function profiles. Serum transaminases and lactic dehydrogenase will be elevated and jaundice may become clinically apparent [7]. If renal or hepatic tests are not evaluated, due to the clinical improvement, the patient may be discharged—with fatal results [13].

The fourth stage, acute liver failure (ALF), or fulminant hepatitis, is characterized by a severe elevation of transaminases, coagulopathy, delirium, headache, hyperbilirubinemia, oliguria, uremia, hepatic encephalopathy, hepatorenal syndrome and acute renal failure [4, 7, 9, 13]. Multi-organ failure, disseminated intravascular coagulation, seizures and death may occur one to three weeks after ingestion. The common factor in patients who will recover is rapid improvement in both symptoms and liver function tests, leading to full recovery [7]. Patients with elevated alanine aminotransferase of 2345–4048 IU/L and aspartate aminotransferase of 2075–3464 IU/L had a significantly higher chance of death. A hepatic coma, developing after a rise in the aminotransferases, also bears a significant risk of death [2, 6]. Overall, the mortality rate varies significantly between 10 and 90% in adults [7].

### Acute liver failure

Fulminant hepatitis is defined as the onset of coagulopathy, and a degree of hepatic encephalopathy within 26 weeks in the absence of underlying liver disease [17–19]. It is a devastating multi-organ syndrome beginning with hepatocellular dysfunction and leading to hemodynamic instability, electrolyte anomalies, renal failure, cerebral edema, encephalopathy and culminating in death [17–19].

The mortality rate of ALF may be as high as 40–50%, depending on the underlying cause (toxin, viral, autoimmune, vascular, metabolic or idiopathic) [18]. The progression of the disease may also be defined as hyperacute (0–7 days), acute (8–28 days), or sub-acute (9–84 days) [20]. There are multiple proposed criteria for urgent liver transplantation (Table 1) [15]. The single set of criteria that has been developed for use in amatoxin poisoning is the Ganzert criteria (Table 2) [7]; however, the most commonly used criteria for determining whether an urgent liver transplantation is needed are the King’s College criteria (Table 3) [7, 15].

**Table 1.** Proposed criteria for urgent liver transplantation in amanitin toxicity

− Clichy criteria
− Ganzert criteria
− Novelli criteria
− King’s College criteria

**Table 2.** Ganzert criteria

1) A decrease in prothrombin time (PT) ≥25% between days 3 and 10 after ingestion
*In conjunction with*
2) Serum creatinine ≥106 μmol/L within the time period of the PT rise

**Table 3.** King’s College criteria

1) Prothrombin time (PT) >100 seconds (INR > .0)
2) At least three of the following:
– PT >50 seconds
– Serum bilirubin >17.5 mg/dL
– Age below 10 years or greater than 40 years
– An interval between jaundice and encephalopathy over 7 days
- Drug toxicity

The King’s College criteria are used to assess the degree of ALF, with a sensitivity of 68–69% and a specificity of 82–92% [18]. In the United States, this instrument has always been questioned as it does not take into account the grade of encephalopathy or the cause of hepatocellular death—the only predictors of outcome [20]. Grade 2 encephalopathy carries a 30% chance of mortality, grade 3 relates to an incidence of 50%, and grade 4 hepatic encephalopathy raised mortality risk to 80% [17]. Grade 3 or 4 encephalopathy is believed to designate irreversible liver damage [18].

## Treatment

### Primary assessment and initial care

No antidote to amanitin is available [11]. There are no randomized, controlled trials for the treatment of mycetismus [17]. In the United States, the mainstays of treatment are supportive. Gastrointestinal decontamination procedures with ipecac and gastric lavage are warranted, although the clinical utility of these is questionable due to the duration of the latency period [1, 7, 11, 21]. The administration of activated charcoal is also warranted, in an attempt to collect any remaining poison within the alimentary tract and excrete it more quickly [11, 16].

Following arrival at an emergency department, blood should be drawn for monitoring of electrolytes and placement of a Foley catheter for strict intake and output measurements. Intravenous access should be established along with fluid resuscitation, with a goal of 1.5–2.0 mL/kg/h urine output for the following four days to augment the renal elimination of the amatoxins. Admission to an intensive care unit is mandatory, for close monitoring of neurological, hemodynamic and respiratory symptoms and management of coagulopathy. Early correction of electrolyte imbalances is imperative and serial monitoring of electrolytes cannot be over-emphasized [7, 15, 21]. Liver function and coagulation tests should be performed serially, to monitor both the stage of the disease and its progression. It must always be kept in mind that, should the patient’s condition continue to deteriorate, transfer to a transplant center is warranted [17, 18, 20, 22]. It is suggested that transfer to a dedicated liver transplant center be mandatory if liver failure is evident during the gastrointestinal and apparent convalescent phases [15, 17].

### Systems-based concerns

In those patients with advanced encephalopathy, intracranial pressure monitoring is advised [18]. In the presence of intracranial hypertension, administration of osmotic agents, mannitol and hypertonic saline, should be followed by therapeutic hypothermia, and induction of a barbiturate coma [18].

Circulatory compromise is common in ALF. This syndrome produces a high-output, low-resistant state [18, 20]. In the presence of hypotension that persists despite adequate volume resuscitation, vasopressers should be administered, with epinephrine being the preferred agent [20]. The correction of coagulopathy is called for; however, administration of vitamin K is useless in ALF. Fresh frozen plasma (FFP) should be given only if there is active bleeding or if the patient is hemodynamically unstable. Bleeding generally occurs from mucosal surfaces, such as the gums or gastric erosions [18, 20]. Recombinant factor VIIa is the primary agent that should be administered for correction of coagulopathy [20].

Acute lung injury occurs in 40% of patients with acute liver failure and contributes to the overall morbidity [17]. A complicating factor, in those patients who develop pulmonary edema or ARDS, is that lung-protective ventilation is problematic, since the increased positive end expiratory pressure can exacerbate cerebral edema and hepatic congestion [17–19]. Infection is common in the setting of ALF and may be as high as 80%, with both fungal and bacterial infections due to dysfunction of the complement system, neutrophils and the Kupfer cells [18, 20]. The infectious processes inhibit the regeneration of hepatocytes and aggressive treatment is indicated [18].

Renal Failure occurs in 50–80% of cases due to several causes: poor perfusion, nephrotoxic substances, sepsis, DIC, or a combination of these [17, 18, 20]; these patients often require continuous hemofiltration [20]. Advances in intensive care have increased spontaneous survival from 15% to 40% [17].

### Chemical therapies

The administration of thioctic acids, hormones, and steroids has been used in the past but these have largely been abandoned [7]. In a retrospective review, Karkhanis *et al*. found that steroids did not offer benefit in the form of overall survival or spontaneous survival in acute liver failure. In addition, they show evidence that administration of corticosteroids decreased the overall survival of patients with elevated model for end-stage liver disease (MELD) scores [23]. Chemical treatment rests with several drugs, the use of which provides hepatocellular protection and increased elimination of amatoxins, as discussed below.

Intravenous vitamin C has been used in acetaminophen and carbon tetrachloride (CCl_4_) poisoning as a hepatocyte protector, by inhibiting hepatic fibrosis caused by lipid peroxidation [22, 24]. Vitamin C is known to be a potent antioxidant and, by this means, can protect the hepatocyte from necrosis by reactive oxygen species produced by the toxin [25]. Cimetidine competitively inhibits of the toxin within the cytochrome P450 system [22]. High-dose intravenous cimetidine is known to inhibit CYP450 enzymes CYP2E1, CYP3A4, and CYP2D6. In animal models, the inhibition of these enzymes has been shown to inhibit hepatocellular necrosis and mitochondrial injury. Patients receiving this treatment have shown decreased levels of ALT and AST, implying a halt to the liver injury. In humans, doses as high as 200 mg/h intravenously have caused no adverse effects [10, 26].

Penicillin G—or benzylpenicillin—is theorized to act in three ways: first, it may bind to plasma proteins (namely albumin), displacing the amatoxins from them and thus allowing their excretion in the urine [10, 14, 22]. The second theory proposes that penicillin G may competitively inhibit the OATP1B3 receptors and prevent uptake by the intestine or liver [10, 25]. Letschert *et al*. reported that benzylpenicillin was able to inhibit amanitin uptake [25]. Finally, gamma-aminobutyric acid (GABA), derived from the intestinal flora, may be incompletely metabolized by the liver due to the toxicity. Benzylpenicillin is believed to decrease the amount of GABA by reducing the amount of intestinal flora which may prevent encephalopathy [27]. Penicillin may be given in doses of 300 000 to 1 000 000 U/kg/d [10, 26]. Dosing usually consists of one million U/kg of penicillin G over the course of a day and 500 000 U/kg per day at a continuous rate for the next two days [15]. Beta-lactams are known to inhibit DNA polymerase alpha, eukaryotic cell proliferation and decrease GABA production, thus limiting hepatic injury when given in large doses. Ceftazidime belongs to the third generation of cephalosporins and may be used as the beta-lactam of choice in lieu of penicillin G; firstly, it is more potent than penicillin G and secondly it has a lower side-effect profile; however, due to the lack of randomized, controlled trials on amatinin poisoning, there is limited data regarding the optimal antibiotic and dosing [25].

N-acetylcysteine (NAC) acts as a glutathione precursor that can be utilized by the body when its natural stores are depleted, so that it is able to bind to the reactive oxygen species created by amanitin [10, 22]. There has been a general trend away from non-specific therapies; however, NAC seems to be the only drug that is the exception to this trend. The reasons behind this seem to be twofold: first, due to the toxic effects from amatoxin, glutathione synthesis is decreased and the addition of NAC provides additional substrate for glutathione production. Second, the reactive oxygen species produces lipoperoxidation of cell membranes, oxidation of proteins, oxidation of nucleic acids and damage to all components within the cell, leading to cell death and glutathione depletion. NAC scavenges free radicals in a non-specific manner, preventing cell destruction by these means [25]. NAC has been shown to increase non-transplant survival and has been proven to be beneficial in grade I–II encephalopathy [19].

Silibinin has been approved for use in Europe since 1982 [8]. Its administration is recommended if the patient presents with symptoms within 48 hours of amanitin ingestion [7]. It is a semi-purified extract of silymarin, a flavolignan derived from the seeds of the milk thistle, and consists of a 1:1 ratio of silibinins A and B [26]. The recommended dose of silibinin is an initial bolus of 5 mg/kg on the first day of treatment, followed by 20 mg/kg by continuous infusion over 24 hours. Treatment should be initiated as expeditiously as possible, since survival has been correlated to the timing of treatment [8]. It has been found that silibinin inhibits the hepatic and intestinal uptake of the toxin by the OATP1B3 receptor system. Through this receptor, the toxin is absorbed by the intestines and transported through the liver, where it is absorbed, a portion is excreted in the bile and the cycle begins again [8].

Silibinin not only promotes hepatocyte growth and inhibition of hepatic oxidation and inflammation, but also demonstrates tissue and metabolic restoring abilities by its ability to stimulate DNA-dependent RNA polymerase I [14, 25, 26]. Its side-effect profile is similar to that of a placebo and there is minimal allergic reaction, most commonly within patients allergic to other aster family plants such as ragweed, daisies, and marigolds [26]. Flushing during intravenous administration appears to be the most common reaction [8]. Unfortunately, this modality of treatment is not commercially available in the United States. A trial was approved by the Food and Drug Administration in 2009 (Clinical trial identifier NCT00915681) and is currently in phase three trials with the dosing of 20 mg/kg/d [28].

The most recent and exciting advance in the treatment of mycetismus by amatoxin was published in September of this year by Garcia *et al.* They describe the use of computer simulation to model the docking of polymyxin B and amatoxin with RNA polymerase II, showing displacement of the amatoxin when polymyxin B is introduced. This simulation was tested in a murine model and 100% survival was found when polymyxin B and amatoxin was administered simultaneously. Those animals that had multiple doses of polymyxin B at 4, 8 and 12 hours following amatoxin exposure had a 50% survival rate. The authors credit the development of the protocol of administering polymyxin B to the computer simulations. Interestingly, the researchers modeled ceftazidime, benzylpenicillin, and silibinin in the computer simulation and showed that these drugs also bind to the same docking structures on RNA polymerase II, but with less affinity than polymyxin B [29].

### Biological replacement therapies

Hemodialysis has never been proven to be beneficial in cases of amatoxin poisoning [26]. Although most cases are anecdotal, a case report of two amanitin poisonings revealed that none of the poison was discovered in the hemodialysis perfusate solution or circuits after treatment [10, 30].

While hemodialysis does not affect outcomes, the emerging therapies of bio-artificial liver, molecular adsorbent recirculation system and single-pass albumin dialysis have had some impact on the treatment of acute liver failure. During times of hepatic disease, the liver function focuses mostly on detoxification. In light of the need for available donor livers and shortage of available organs, extracorporeal devices have been designed to function as detoxification units for the diseased liver. The liver support devices mentioned above provide a bridge from fulminant hepatitis to the time when transplantation becomes possible [19]. These devices also function to allow for an environment for hepatocyte regeneration [20].

Unfortunately, the vast majority of reports on the functional efficacy of these devices are based on case series or case reports [20]. These reports have issued no survival benefit in respect of ALF; however there does appear to be an improvement in the degree of encephalopathy [18, 20]. A total of twelve randomized, controlled trials have been performed and these have twice been reviewed by meta-analyses: no benefit beside improvement in the encephalopathy has been discovered [19].

### Transplantation

As stated above, a low threshold must be maintained for transferring patients to a transplantation center, since orthotopic liver transplantation (OLT) is the only definitive treatment for patients with ALF [18]. Should no exclusion criteria be met, (Table 4). OLT is an effective and well established procedure for patients suffering from ALF, that has been proven to increase survival, although it has never been scrutinized by randomized, controlled trial [19, 20]. ALF accounted for 7% of the indications for liver transplantation in the United States from 1999 through 2008; mycetismus-induced ALF accounts for 5–12% of all liver transplantion activity [19, 31].

**Table 4.** Exclusion criteria for orthotopic liver transplantation

− Substance abuse
− Suicidal predilection
− Psychiatric disorder
− Uncontrollable sepsis
− Irreversible brain damage
− Extrahepatic malignancy
− Cardiovascular failure requiring more than 1 mcg/kg/min norepinephrine infusion
− ARDS requiring >60% FiO_2_ and positive end expiratory pressure >12cm H_2_O

When liver transplantation is performed, survival is spontaneously increased from 40% to 60% (overall cases) [17]. In addition to whole-organ deceased donor transplantation, living-donor liver transplantation is a viable option, but it must be remembered that donation carries a 0.2% risk of death from the procedure [20]. The majority of deaths occur during the first year after transplant, 77% of fatalities. Of those mortalities that occur, the highest incidence is within the first three months following transplant, approximately 86% of deaths occur during this time [31]. Overall patient survival rates in Europe at 1, 3, 5, and 10 years were 74%, 70%, 68%, and 63%, respectively [20, 31]. The United States has slightly better outcomes, with one-year survival at 82% [31].

Few studies have been made with the purpose of evaluating treatment during amatoxin intoxication. Ganzert *et al*., with an understanding of the uncertainty over which patients must have liver transplantation, took upon themselves the task of developing criteria for liver transplantation in this setting (Table 2). It was determined that decreases of both prothrombin (prothrombin index less than 25%) and serum creatinine were more specific than transaminase levels or serum bilirubin for determining the need for transplantation. Age was also found to have no influence on predictive status [32].

Escudie *et al*. contradicted this study by demonstrating that the Clichy criteria (Table 5) and Ganzert criteria had similar sensitivities—approximately 85%—while utilization of the King’s College criteria (regardless of whether acetaminophen poisoning or non-acetaminophen poisoning criteria were used) had sensitivity, specificity and accuracy of 100% predictive value for those patients requiring liver transplantation [12]. A novel approach to determining whether a patient will require liver transplantation was proposed by Novelli *et al.* Their research produced six influential factors: ICP, IL-6, systemic vascular resistance indices, percentage of the reduction of lactate, GCS £9, and the fact that the number of molecular adsorbent recirculation system treatments could be predictive of the need for transplant [33].

**Table 5.** Clichy criteria

1) Grade 3–4 hepatic encephalopathy
2) Either: a) Age <30 years and Factor V levels <20% or b) Age >30 years and Factor V levels <30%

### Long-term prognosis

The prognoses of patients suffering from mycetismus are extremely variable. Much of the research concludes that, the earlier the treatment is initiated when amanitin poisoning is suspected, the better the results; for example, the murine model of amatoxin and polymyxin B has shown that those animals treated concurrently with both agents have a 100% survival rate, while those treated 4 hours later have a 50% chance of survival [29]. Liver transplantation is usually a last-ditch effort for treatment, but has been shown to improve survival in those suffering from ALF secondary to amatoxin [17, 19, 29, 32]. Patients treated in an intensive care unit fare better, probably due to the ability to monitor for and treat systems-based and systemic complications early [18, 21]. Those patients who suffer from acute lung injury, infection, cardiovascular compromise or any of the aforementioned complications fare worse [21]. Overall, the survival rates for adults range from 10–90% [7]. Those patients who undergo liver transplantation for acute liver failure have poorer survival rates than those who receive liver transplantation due to chronic liver failure; however, one-year survival rate ranges from 65–80% [19].

## Conclusions

Mycetismus represents an unusual but significant diagnostic challenge in addition to being a difficult entity to treat, with a variable clinical course. Whilst significant advances have occurred in intensive care to increase the chances of survival, the need for liver transplantation will remain high should the patient progress to acute liver failure. Although support with chemotherapeutics increases survival, artificial detoxification by extracorporeal means and transplantation will be necessary. With the confounding picture of the seemingly improved patient following the gastrointestinal phase, a diagnosis may be overlooked, resulting in death.

For these reasons, we stress that a centralized agency should be charged with the recording of data and researching improved means of survival following ingestion of the toxins. An algorithmic system of treatment has not yet been established due to the lack of prospective clinical trials. Hope exists with the discovery of the effects of polymyxin B on amatoxin and RNA polymerase II, but its effects in human subjects has yet to be tested. We must repeat that, due to a lack of response to calls for research into the treatment of this disease, silibinin has been unused in America for the past three decades. Through the use of a centralized agency, it would be possible to determine when management such as transplant, liver support devices, medications, and system specific therapies might be the most effective, and to formulate the continued evolution of management in a controlled fashion.

Funding: No funding was received by either author in respect of the above research.

*Conflict of interest statement*: none declared.
